# Protein Unfolding: Denaturant vs. Force

**DOI:** 10.3390/biomedicines9101395

**Published:** 2021-10-05

**Authors:** Colleen Kelly, Matthew J. Gage

**Affiliations:** 1 Department of Chemistry, University of Massachusetts Lowell, Lowell, MA 01854, USA; colleen.kelly@med.uvm.edu; 2 UMass Movement Center (UMOVE), University of Massachusetts Lowell, Lowell, MA 01854, USA

**Keywords:** protein refolding, chemical denaturation, thermal denaturation, magnetic tweezers, titin, immunoglobulin domain

## Abstract

While protein refolding has been studied for over 50 years since the pioneering work of Christian Anfinsen, there have been a limited number of studies correlating results between chemical, thermal, and mechanical unfolding. The limited knowledge of the relationship between these processes makes it challenging to compare results between studies if different refolding methods were applied. Our current work compares the energetic barriers and folding rates derived from chemical, thermal, and mechanical experiments using an immunoglobulin-like domain from the muscle protein titin as a model system. This domain, I83, has high solubility and low stability relative to other Ig domains in titin, though its stability can be modulated by calcium. Our experiments demonstrated that the free energy of refolding was equivalent with all three techniques, but the refolding rates exhibited differences, with mechanical refolding having slightly faster rates. This suggests that results from equilibrium-based measurements can be compared directly but care should be given comparing refolding kinetics derived from refolding experiments that used different unfolding methods.

## 1. Introduction

The seminal work of Christian Anfinsen and Cyrus Levinthal [1,2] initiated what is now over half a century of work to decipher the principles that govern how a protein transitions from an extended linear state to its final, active conformation. The majority of this work has relied on denaturing a purified protein and exploring the conformational changes that allow it to return to its native state [3,4,5]. This approach has established the majority of the core principles of protein refolding and continues to provide new insights into this crucial and complex process. While bulk refolding studies have provided the foundation for our understanding of protein refolding, the advent of single-molecule refolding approaches as well as the development of techniques that allow monitoring of protein folding both in vivo and on the ribosome has provided an even greater understanding of how a newly formed protein finds the correct conformation [6,7,8].

A critical step in protein refolding studies is the denaturation of the purified protein, which is most commonly accomplished either by chemical denaturants or by thermal denaturation. These two approaches disrupt existing protein structure differently and so it has often been hypothesized that different unfolding methods result in different unfolding states due to differences in the types and number of intramolecular contacts existing in the unfolded state [9,10,11]. For example, chemical denaturants such as urea form hydrogen bonds with the peptide backbone and disrupt the core stabilizing interactions in the protein [12]. Alternatively, when a protein is thermally unfolded, the increased energy in the system disrupts many of the stabilizing interactions in the protein but some native and non-native hydrophobic interactions may be maintained or even strengthened at elevated temperatures [13,14]. It is these differences that established the hypothesis that refolding might be initiated at different points on the folding funnel based on the method of denaturation.

In recent years, a growing number of studies have applied force as a mechanism to unfold the protein of interest [15,16,17,18,19,20]. In these studies, a sufficient level of force is applied so that the protein rapidly unfolds and then force is relaxed, allowing the protein to refold. The seminal work in this area was done in the mid-1990s, where immunoglobulin domains from the muscle protein titin were stretched and unfolded using atomic force microscopy (AFM) [21,22,23,24]. Since those initial studies, numerous other proteins have been studied, not only by AFM but other force spectroscopy techniques such as optical trapping and magnetic tweezers. Many elastic proteins such as titin undergo unfolding and refolding as part of their function, so force spectroscopy-based folding studies provide a physiologically based method to study the transition between the folded and unfolded state.

One of the most studied proteins by force spectroscopy has been the titin protein, which has over 90 unique immunoglobulin (Ig) domains. One of the first domains from this protein to be characterized was the I91 Ig domain (originally called I27) [18,21], though since then a variety of Ig domains have been characterized using both force spectroscopy and more traditional bulk refolding studies using either CD or intrinsic tryptophan fluorescence since those initial studies. The variety of techniques that have been used to characterize refolding of titin domains raises the question of whether it is possible to correlate results from folding studies that utilized different techniques. Botello et al. used the I91 domain to compare mechanical unfolding by AFM with chemical unfolding and demonstrated that both techniques resulted in similar unfolded states [25]. In a similar study using the same domain, Carrion-Vasquez showed that the unfolding rate of a tethered homopolymer extrapolated to zero force was the same as the chemical unfolding rate extrapolated to zero denaturant [26]. These studies suggested that it is possible to apply results from bulk refolding experiments to mechanical unfolding/refolding studies.

We have been using the I83 domain, the terminal C-domain in the N2A region of titin, to further explore the question of how closely single molecule folding experiments correlate with chemical and thermal denaturation studies. Previous work by our lab has demonstrated that while the I83 domain is structurally similar to the I91 domain, it has a free energy of unfolding that is 2–3 times lower than I91 [26,27]. We have also previously demonstrated that this domain refolds reversibly and the ΔG_refolding,_ as determined by chemical denaturation, is equivalent to the ΔG_unfolding_ [4,27]. Most interesting, the stability of this domain is increased in the presence of calcium due to binding of calcium to a non-canonical calcium binding site along the I82-I83 interface [27]. These unique characteristics and the lack of mechanical characterization of this domain makes this an interesting system to explore the relationship between different refolding methods.

We chose to use magnetic tweezers to explore the mechanical unfolding and refolding pathway of I83 for two primary reasons. First, it provides the ability to monitor discrete unfolding and refolding events at physiologically relevant forces (<20 pN) with high resolution [17] without the photobleaching effects that can occur when using optical tweezers [28]. Recent studies have demonstrated that there is a conformational equilibrium between folded and unfolded states of Ig domains that are held for extended periods at low forces (6–10 pN) [29,30,31], so the ability to measure these transitions without damage to the protein has provided insights that are not obtainable through other approaches. Second, the lack of damage to the sample during the measurement allows multiple, repeated measurements to be made on the same molecule as buffer components are added or exchanged through the flow cell. This makes magnetic tweezers the ideal force spectroscopy tool for this study.

The focus of this work was to correlate the thermodynamic and kinetic properties of the I83 domain as measured following chemical, thermal, and mechanical unfolding. Our results demonstrate that the ΔG_unfolding_ and the ΔG_refolding_ are statistically equivalent regardless of the approach used for unfolding. In contrast, mechanical unfolding exhibited faster unfolding kinetics and faster refolding kinetics than chemical denaturation kinetics. We also demonstrate that including calcium at pCa ~4.3 stabilizes the I83 domain in force spectroscopy experiments, consistent with previously observed stabilization in chemical and thermal denaturation experiments. Most interestingly, this stabilization is observed under weak forces but not at higher forces, denaturant concentrations, or temperatures. This suggests that the calcium binding site disappears as more of the overall structure of the domain is lost. Overall, our results demonstrate that all three unfolding approaches tested are thermodynamically equivalent, but they are not kinetically equivalent.

## 2. Materials and Methods

**Reagents:** Chemicals were obtained through standard chemical suppliers such as Fisher Scientific, Waltham, MA, USA and Sigma-Aldrich, St. Louis, MO, USA. The coding sequences for the three Ig domains were codon optimized for *Escherichia coli* and synthesized by GeneArt. The synthesized genes were subcloned into the pET151-D-TOPO vector for expression. The NCBI sequence for the N2-A isoform of titin (NP_035782) was used as the reference for the sequences of the individual Ig domains.

### 2.1. Expression and Purification of the Individual I83 Domain and Halo-(I83)_6_ Polymer

Expression and purification of the monomeric I83 domain was as published in our previous work [27]. Expression plasmids for Halo-(I83)_6_ containing an N-terminal Halo-tag and C-terminal Avi-tag were transformed into chemically competent *Escherichia coli* BL21 (DE3) cells. Autoinduction media cultures were inoculated by 0.5% (*v*/*v*) overnight cultures and grew at 30 °C and 230 rpm for 16–18 h. Purification of Halo-(183)_6_ followed the same method as for the monomeric I83 [27], sans cleavage of the HIS-tag for Halo-(183)_6_. Size exclusion chromatography fractions containing purified Halo-(183)_6_ were pooled and dialyzed into storage buffer (20 mM HEPES, 138 mM KCl, 12 mM NaCl, pH 7.4) with 20% glycerol, flash frozen, and stored at −80 °C.

### 2.2. Chemical Stability

Purified protein was diluted to a final concentration of 100 µg/mL in storage buffer (+/− 50 µM Ca^2+^) with varying urea concentrations (0–7.6 M urea). Samples were placed in a quartz 96-well plate and incubated at room temperature (22 ± 2 °C) for one hour prior to data collection to ensure equilibrium was reached. Samples were excited at 280 nm and the emission spectra were collected in 2 nm steps from 300 to 450 nm at 25 °C using a SpectraMax M3 plate reader. The Center of Mass (*CoM*) of each spectrum was calculated using Equation (1), where *I* is intensity and $\overset{-}{v}$ is the wavenumber:

(1) $$CoM=\frac{\Sigma \left(\overset{-}{v}\cdot I\right)}{\Sigma I}$$

CoM was plotted as a function of urea concentration and fit using linear extrapolation [32] to determine the Δ*G*_unfolding_ using Equation (2)

(2) Keq= e−ΔG+mxRT

where *x* is the concentration of urea. A two-way *t*-test analysis of variance was used to determine statistical significance using a value of *p* < 0.01 as the cutoff for significance.

### 2.3. Thermal Stability

Circular Dichroism (CD) spectra of 100 µg/mL samples of the purified and I83 domains were measured using a JASCO J-1500 CD Spectrophotometer (JASCO International Co., Tokyo, Japan). Variable temperature measurements were made at 222 nm and 208 nm every 0.1 °C nm from 15–95 °C while samples were placed in a sealed quartz cuvette with a 0.1 cm path length and heated at a rate of 1 °C/min using a Peltier temperature controller. The signal (mdeg) was converted to molar ellipticity (*θ*) using Equation (3):

(3) $$\theta =\frac{mdeg\cdot M}{C\cdot L\cdot 10}$$

in which *M* is the molecular weight, *L* is the path length of the cell in centimeters, and *C* is the concentration of the protein in g/L. Values reported are the average of four scans and were background subtracted before deconvolution.

The free energy for the two-state refolding processes were determined by using a derivation of the Gibbs-Helmholtz, in which the specific heat capacity (Cp) of the folded and unfolded state are assumed to be the same [33]. CD data were fit to an unfolding curving that determined the enthalpy (Δ*H°_m_*) and unfolding midpoint (*T*_m_) using Equation (4):

(4) $$\Delta G^{{}^{\circ}}\left(T\right)=\Delta H^{{}^{\circ}}{}_{m}\left(1-\frac{T}{T_{m}}\right)$$

where *T* is the melting temperature of the protein, such that the midpoint temperature (*T*_m_) yields a free energy of zero [33].

### 2.4. Chemical Folding Kinetics

Unfolding kinetics were measured by the addition of various concentrations of urea to achieve a final protein concentration of 100 µg/mL. Samples were excited at 280 nm and the emission spectra were collected at 20 °C. The unfolding emission spectra were monitored at 354 nm every 3 s using a SpectraMax M3 plate reader and rate constants were determined by fitting data to a single exponential using Equation (5):

(5) $$y=c+A\left(1-e^{-\mathrm{kt}}\right)$$

in which *y* in the signal intensity, *c* is the *y*-intercept, *A* is the amplitude of the curve, k is the rate constant, and t is time in seconds.

### 2.5. Flow Cell Preparation

Magnetic tweezers measurements were performed in fluid chambers that were functionalized by (3-aminopropyl)-trimethoxysilane in ethanol (1% *v*/*v*, Sigma-Aldrich, St. Louis, MO, USA), and glutaraldehyde (1% *v*/*v*, Sigma Aldrich) as described by Popa et al. [17]. HaloTag amine (O4) ligand (Promega, Madison, WI, USA) was added to create an attachment point for the protein of interest. Halo-(I83)_6_ protein was diluted in 20 mM HEPES buffer and added to a functionalized flow cell. Chambers were washed with TRIS buffer and then streptavidin coated paramagnetic beads (Dynabeads M-270, Invitrogen, Waltham, MA, USA) were added to attach the biotinylated C-terminus of the protein.

### 2.6. Single-Molecule Measurements

A home-built magnetic tweezer system modeled after those systems used by the Fernandez Laboratory at Columbia University [17] was used for force-induced unfolding and refolding measurements. The system was calibrated using Protein-L and the fitting parameters of the magnet law (Equation (6)) for the system are “a” = 177 ± 17 pN and “b” = 1.07 ± 0.05 nm [17].

(6) $$F=a\cdot e^{-b\cdot MP}$$

A Z-stack library of both a protein-tethered paramagnetic bead and a fixed reference bead were initially acquired prior to each measurement and this was used to calculate the real-time Z-position displacements using the image processing method developed by the Fernandez Lab [17]. The extension of Halo-(I83)_6_ in nanometers (nm), as induced by applying pulling forces in the piconewton (pN) range, was measured vs. time using an imaging rate of 1800–2000 frames per second (fps). The load rate of the magnetic force was negligible (<0.01%) as compared to the overall unfolding rates observed. Noise-reduction was applied using the AutoStepFinder fitting algorithm in MatLab (R2021a), developed by Kerssemaker [34]. Five independent measurements were made at each force based on previous studies that have demonstrated that five single-molecule measurements provide a reliable mean that is within one SD of the population mean [35].

### 2.7. Force-Induced Folding and Refolding Kinetics

The rate of homopolymer unfolding and refolding was determined using the same single exponential equation sed for the fluorescent kinetic experiments to fit the stepwise unfolding and refolding events over time when initiated by a change in force (Equation (5)). Force-induced folding comparisons of Halo-(I83)_6_ in absence and presence of calcium were conducted by exchanging the buffer components in the flow cell while taking measurements on a single molecule.

### 2.8. Comparison of Mechanical and Chemical Stability and Kinetics

Mechanical and chemical stabilities were compared by substituting unfolding (*ku*) and refolding rates (*kf*) in for the equilibrium constant (*K)* in the free energy equation, as shown in Equation (7) using 293 K as T.

(7) $$\Delta G=-RT\;ln\frac{ku\left(f\right)}{kf\left(f\right)}$$

Comparison of chemical and mechanical unfolding rates by chemical and mechanical means was done using a chevron plot. The mechanical unfolding rate of a single protein domain in a homopolymer was compared to that of a solution containing the individual domains by extrapolating to zero force and 0 M urea, as in previous studies [26].

## 3. Results

### 3.1. Thermal and Chemical Equilibrium Studies Yield Similar Stabilities

**Figure 1:**
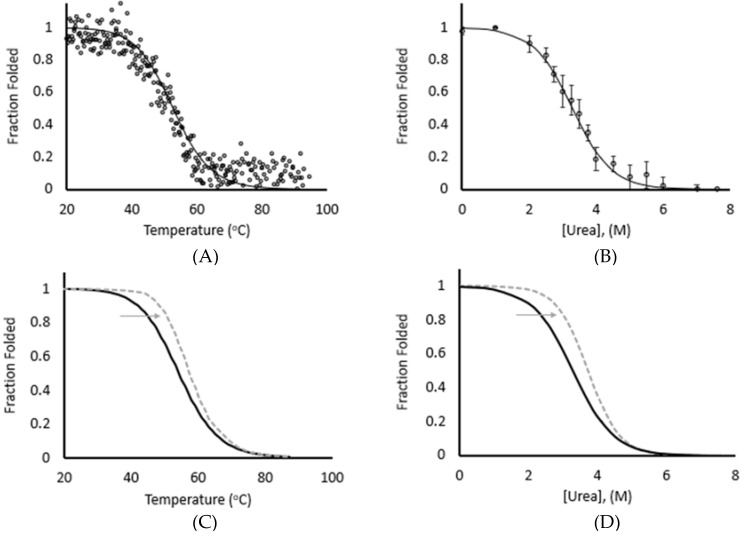
Thermal and chemical unfolding of I83 yield similar free energies and calcium stabilization. (**A**) CD data obtained by thermal melt for I83 (○) is fit by a two-state unfolding model (solid line) with a thermal midpoint (T_m_) of 53.2 °C. (**B**) Center of mass calculated from tryptophan fluorescence spectra measured during chemical unfolding (○) is fit by a two-state unfolding model, with a midpoint ([Urea]_50%_) of 3.2 M. Error bars show ± one standard deviation. (**C**) Thermal unfolding fit of I83 in the absence of calcium (black) is overlayed with the unfolding fit at pCa 4.3 (dashed gray line). (**D**) Chemical unfolding fit of I83 in the absence of calcium (black) is overlayed with the unfolding fit at pCa 4.3 (dashed gray line). Reprinted from Reference [4].

Protein refolding experiments generally start by unfolding the protein using either chemical or thermal denaturation or by the application of mechanical force [36,37]. Each method unfolds the protein differently and it has been hypothesized that different methods result in different unfolded states. If true, comparing refolding results derived using different unfolding methods could lead to incorrect conclusions about the folding pathway utilized by a protein under certain condition [38,39]. We used the I83 immunoglobulin-like (Ig) domain from the N2A region of the muscle protein titin as a model system to compare the stability following chemical, thermal, and mechanical unfolding to explore this hypothesis. Chemical stability was measured using intrinsic fluorescence and the free energy was calculated using linear extrapolation [33]. Thermal stability was measured by Circular Dichroism (CD) and the change in the molar ellipticity vs. temperature was fit using a derivation of the Gibbs-Helmholts equation [40]. As previously published, the thermal ΔG_unfolding_ for this domain is 3.2 ± 0.1 kcal/mol (Figure 1A) and the ΔG_refolding_ is 3.1 ± 0.2 kcal/mol, demonstrating that this is a reversible process (Table 1, [4]). The ΔG_unfolding_ from chemical denaturation is 3.31 ± 0.3 kcal/mol while the ΔG_refolding_ is 3.40 ± 0.04 (Figure 1B, [4]). There is not a statistically significant difference between the ΔG_unfolding_ for both processes but there is a statistically significant difference between the ΔG_refolding_ from chemical and thermal unfolding (*p*-value < 0.05). This suggests that, while the energy requirements to unfold the protein are similar from either chemical or thermal denaturation, there is a subtle difference in the overall nature of the unfolded state that decreases the free energy requirement of refolding following thermal denaturation [41,42].

Previous work for this domain has demonstrated increased stability in the pres-ence of Ca^2+^ due to a non-canonical calcium binding site involving the F–G loop [4]. In both thermal and chemical denaturation studies, this results in an increase in the ΔG_unfolding_ of 1.0 kcal/mol at pCa 4.3 (Figure 1C,D). The *m*-value, representing the slope of the transition from the folded to the unfolded state, increased by 33% in both cases as well. This suggests that the observed increase in stability is due to increased coop-erativity from binding of calcium to the domain [27].

### 3.2. Force Unfolding and Refolding of Halo-(I83)_6_ Shows Stabilization in Calcium

**Figure 2:**
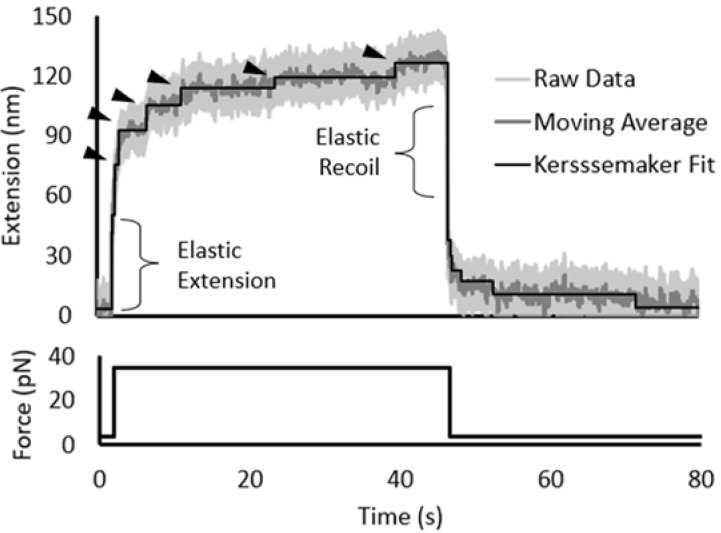
Step-fitting of 35-pN unfolding of (I83)_6_ polymer. Unfolding data demonstrated system noise of approximately ± 20 nm (light gray). A moving average (dark gray) and Kerssemaker fit (black line) more clearly illustrate the extension, unfolding, and refolding of the hexamer. The applied force over time, shown below the data trace, started at 4 pN, was stepped to 35 pN, and then returned to 4 pN after ~45 s. The unfolding steps of the individual Ig domains are marked by triangles and follow the initial elastic extension of the polymer.

Proteins have an unfolding “fingerprint” that is defined as the minimum force and time that produces an extension step for each domain in a polymer chain. Previous studies on other titin Ig domains have shown that I91 has an unfolding fingerprint of 102 pN over 100 s while the fingerprint for the less stable I10 domain is 64 pN over 10 s [31]. The ΔG_unfolding_ of the I91 domain is 2–3× greater than that of the I83 domain when measured using chemical unfolding [26,27] so it was hypothesized that I83 might have similar mechanical stability to the I10 domain and unfold at forces ranging from 30–60 pN. To test this hypothesis, flow cells were prepared with a Halo-(I83)_6_ construct attached to the surface and the attached protein was unfolded using magnetic tweezers. Individual proteins were identified by the slight movement of the magnetic bead and a 4 pN force was applied to the system to reduce Brownian motion. Unfolding was induced by stepping the magnetic force from 4 pN to a range of higher forces. As shown in Figure 2, unfolding occurs within 40 s of stepping the force to 35 pN and the six unfolding events representing the individual I83 domains unfolding can be observed after the initial characteristic elastic extension step. The average extension step is 15.9 ± 1.2 nm, which is consistent with the extension length of a typical Ig domain at that force [30,43]. When the force is dropped from 35 pN back to 4 pN, an elastic recoil is observed followed by a series of refolding steps. Both elastic extension and recoil observed in our experiments is consistent with unfolding profiles observed with other multi-Ig polymers [30].

**Figure 3:**
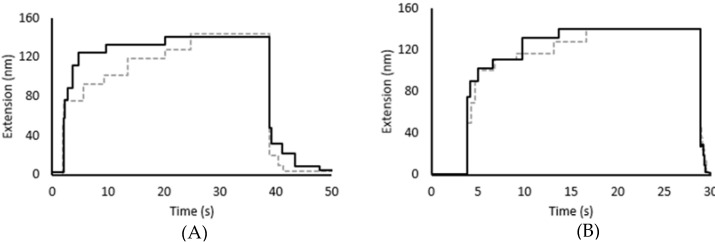
Sample unfolding plots from magnetic tweezers exhibit slower unfolding rates in presence of calcium. (**A**) Unfolding steps at 40 pN occurred more rapidly in the absence of calcium (black) than in the presence of calcium (dashed gray). (**B**) Unfolding steps at 50 pN occurred slightly more rapidly in the absence of calcium (black) than in the presence of calcium (dashed gray). The first unfolding step is not visible when unfolding at 50 pN because it occurs during the elastic extension step.

The unfolding time for the hexamer was measured at a range of forces to determine the relationship between force and unfolding rate. The average time to unfold all six domains at 35 pN of force is 41.2 ± 9.5 s. This decreases to 20.5 ± 11.4 s at 40 pN and 14.8 ± 12.2 s at 50 pN (Figure 3), demonstrating that the rate of unfolding is correlated with the applied force, as would be predicted. Previous studies on other titin Ig domains have reported unfolding on this time scale at forces of 50 to 100 pN, depending on the stability of the domain [30,31,43]. The lower unfolding force range observed for I83 is consistent with the lower stability of this particular Ig domain as observed in both chemical and thermal denaturation experiments.

As calcium has been shown to increase the stability of I83 in equilibrium experiments, we predicted that a similar effect would be observed with mechanical unfolding. The unfolding time for the complete hexamer was increased in the presence of calcium, as was observed previously for both the chemical and thermal unfolding experiments. The average unfolding time for this domain at 35 pN increased from 41.2 ± 9.5 s to 55.6 ± 8.2 s in the presence of calcium. Calcium also increased the unfolding time from 20.5 ± 11.4 s to 32.4 ± 9.9 s at 40 pN (Figure 3A) though interestingly there was not a similar statistically significant stabilization observed at 50 pN (14.8 ± 12.2 s to 17.9 ± 11.5 s, Figure 3B). This suggests that the interdomain interactions that are enhanced by calcium are easily ruptured by applying a 50 pN force. The actual calcium concentration is not known in these experiments since it is not possible to calculate the degree of dilution of the calcium during the buffer exchange step, but it is estimated to be approximately pCa 4. The change in unfolding rate with calcium present at 35 and 40 pN is ~30%, which is consistent with the change in unfolding rate observed in chemical unfolding experiments [27].

**Figure 4:**
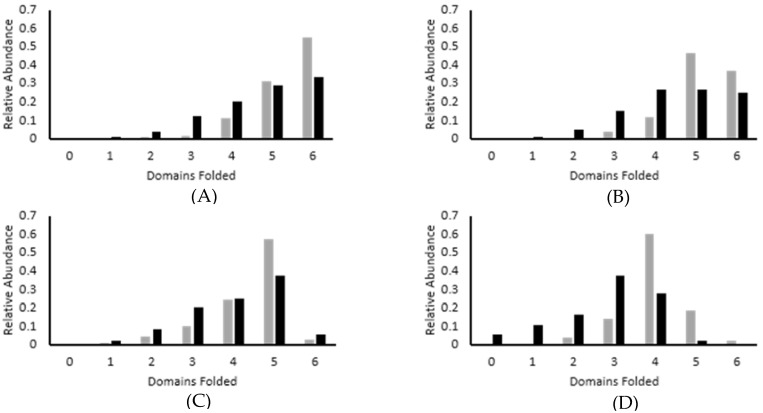
Sample refolding traces of (I83)_6_ polymer in calcium shifts equilibrium to folded state. The same molecule was monitored in absence (black) and presence (gray) of calcium. A histogram is used to show the distribution of the number of folded domains as measured by extension values over 60 s. A shift toward the folded state is observed in the presence of calcium at (**A**) 4 pN, (**B**) 6 pN, (**C**) 8 pN, and (**D**) 10 pN.

To further explore folding at low forces, refolding experiments were conducted by extending the polymer fully at 75 pN for 10 s to fully unfold the protein and then stepping the force down to between 2 and 10 pN for 60 s to monitor both the rate of folding and the ratio of folded to unfolded domains following the 60 s recovery period (Figure 4). The degree of refolding directly correlates with the applied force, as would be expected. The fastest refolding occurs at 4 pN (Figure 4A), as evidenced by this force producing the highest ratio of folded to unfolded domains, followed by 6 pN (Figure 4B), 8 pN (Figure 4C), and 10 pN (Figure 4D) respectively. These results are consistent with similar studies that have been conducted on other Ig domains [29,30,31].

**Figure 5:**
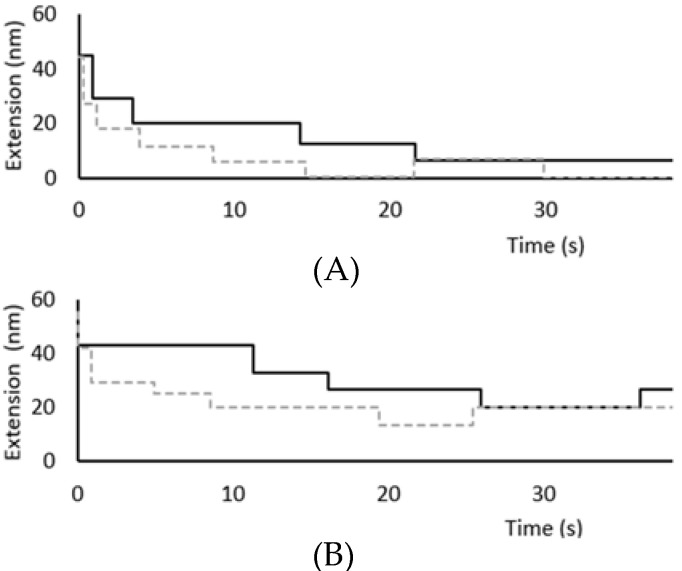
Refolding Traces of (I83)_6_ polymer at 4 pN and 10 pN. Sample unfolding traces at (**A**) 4 pN and (**B**) 10 pN of force illustrate faster and more dynamic refolding in presence of calcium (dashed gray) vs. the absence of calcium (black) as demonstrated by a greater decrease in extension and more frequent folding transitions over time.

This experiment was repeated in the presence of calcium to determine if calcium impacts the rate of I83 refolding in mechanical experiments as has been observed in chemical denaturation experiments. Two important differences were observed between refolding in the presence of calcium compared to refolding in the absence of calcium. First, the domains appear to refold more quickly in the presence of calcium. In a sample refolding trace, the first refolding events were observed immediately in the presence of calcium and began about 1 s later in the absence of calcium when the force was decreased to 4 pN (Figure 5A). Similarly, the first refolded domains were observed in 2 s after the force was decreased to 10 pN in the presence of calcium, compared to nearly 10 s in the absence of calcium (Figure 5B). A similar pattern was observed at the other forces tested as well. The second observation is that the number of unfolding and refolding events observed over the same time period was greater in the presence of calcium, suggesting that calcium shifts the equilibrium toward the folded state, which is most apparent at 10 pN (Figure 5B). At this force, half of the domains remain unfolded in the absence of calcium, whereas 4 of 6 domains refold after the same recovery time in the presence of calcium (Figure 4D). Our current working model is that calcium helps to stabilize an intermediate in the folding pathway as well as stabilizing the final folded structure, shifting the equilibrium toward the folded state. This model explains why calcium impacts both the folding kinetics and the overall stability of the domain and we are working to further test the validity of this model.

### 3.3. Chemical and Mechanical Folding Yield Similar Rate Constants and Free Energy Values

**Figure 6:**
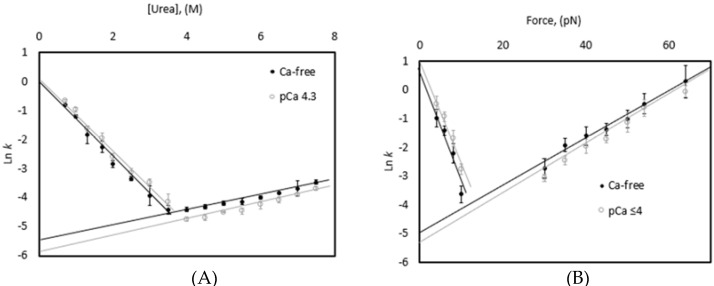
Chevron plots for I83 yield similar, but different rate constants at zero denaturant and zero force. (**A**) The natural log of unfolding rate constants for urea denaturation yielded slower unfolding at pCa 4.3. Average values (N = 4) in the absence of calcium (black) and in the presence of calcium (gray) are shown ± one standard deviation. (**B**) The natural log of force-induced unfolding and refolding rates illustrate slower unfolding between 30 and 40 pN and fast refolding at force ≤10 pN. Average values (N = 5) in the absence of calcium (black) and in the presence of calcium (gray) are shown ± one standard deviation.

The addition of calcium appears to shift the equilibrium toward the folded state at forces ≤40 pN, suggesting an increase in the ΔG_unfolding_ in the presence of calcium. To explore this hypothesis, a comparison of folding rates from chemical denaturation and force was conducted in the presence and absence of calcium. Rate constants were determined for both unfolding and refolding processes at a range of both urea concentrations and pulling forces. Each experiment was analyzed using a nonlinear, least squared fit and the results were averaged to generate a Chevron-plot (Figure 6, Table 2). The k_o_ for both unfolding and refolding was determined by extrapolating to zero denaturant using a linear fit. These rate constants were used to calculate the ΔG_unfolding_ for both chemical and mechanical unfolding.

A statistically significant difference is observed between the rate of chemically induced unfolding and mechanically induced unfolding (Table 2), with chemical unfolding being slower (0.004 ± 0.0004 s^−1^ to 0.0067 ± 0.0019 s^−1^, respectively, *p* value < 0.05). A similar shift was observed for refolding as well, with refolding occuring faster following mechanical unfolding (2.72 ± 0.66 s^−1^ to 0.99 ± 0.13 s^−1^). Interestingly, since there is a comparable shift to both the folding and unfolding rates, there is not a statistically significant difference in the ΔG_unfolding_ (3.3 ± 0.25 (chemical) to 3.5 ± 0.62 (mechanical), *p*-value = 0.56). These experiments were conducted at pCa ~4.3 and a similar effect was observed. The impact of calcium appears to be greatest at mid-range denaturant concentrations (3–5 M urea) or forces (10–30 pN), where calcium is likely shifting the equilibrium toward the folded state. This is illustrated by the minima of the chevron plot for experiments conducted in the presence of calcium being shifted toward a higher concentration of either denaturant or force. Calcium reduces the extrapolated natural log of the rate of unfolding in the presence of denaturant and had no significant impact on refolding in the presence of denaturant. It is interesting to note that, while calcium impacts both the folding and unfolding in the presence of force, there is a larger change in the extrapolated natural log for the refolding rate, suggesting that either calcium is impacting the folding process more strongly than the unfolding process or that calcium has more of an impact in the presence of the low forces used to measure refolding.

## 4. Discussion

Single-molecule force-based refolding studies offers unique insights into the folding pathway of individual molecules, which differs from the insights that can be gained in bulk refolding studies initiated from a chemically or thermally denatured state. Bulk refolding experiments have the advantage of being relatively easy to conduct and can generally be done with equipment available in most laboratories. It is also relatively easy to alter variables such as temperature and buffer conditions in these types of experiments. These types of studies have provided valuable insights into protein folding over the years and have shown relatively good correlation with more recent single-molecule experiments [3,9,44,45]. Single-molecule fluorescence, optical trapping, atomic force microscopy and magnetic tweezers are all types of single-molecule techniques that have become more generally accessible and which have provided new and exciting insights into the folding and equilibrium dynamics of single molecules [15,18,19,28,31,46,47,48]. Despite the growing body of single-molecule refolding studies, there have been a limited number of studies correlating results from bulk refolding experiments with single-molecule results, which is the focus of this study.

Immunoglobulin domains make an ideal model system to explore the question of correlation between single-molecule and bulk solution measurements. There is a growing body of work exploring the physiological role of force-induced unfolding and refolding events in mechanical proteins such as titin that have repeated Ig-domains [20,31,43,49,50]. It has been found that Ig domains, like I10 and I91, are much more dynamic than initially believed and have been shown to undergo unfolding and refolding transitions at physiologically relevant forces (≤10 pN) suggesting that these types of transitions could impact titin elasticity in vivo [30,31,43,51]. These studies have highlighted the importance of using mechanical assays capable of generating small forces to study the folding equilibrium of mechanical or elastic proteins [31]. AFM has provided many valuable insights into molecular stability but it is not capable of reaching forces in the range 5–15 pN due to the size and relatively high stiffness of the cantilever [28]. It is also not currently possible to watch the dynamics of a molecule over time with AFM. In contrast, magnetic tweezers offer a powerful alternative that is capable of achieving forces from approximately 1 pN to over 200 pN. This dynamic range can be achieved by applying very small changes in magnetic field strength through small changes in the magnet position [28]. Moreover, monitoring unfolding and refolding over a range of small forces and over time can offer unique mechanistic insight into the physiologically relevant folding events taking place.

### 4.1. Correlations Observed across Methods

The focus of this study was to compare both the ΔGs and rates of both folding and unfolding derived using chemical, mechanical, and thermal denaturation. Our results demonstrated that there is not a statistically significant difference between the ΔG_unfolding_ and ΔG_refolding_ regardless of the method of denaturation. This suggests that it would be possible to draw correlations between free energies reported in different studies, even if the studies relied on different methods of denaturation. This is significant since techniques such as intrinsic fluorescence are faster and easier than single-molecule techniques and can be used in some cases in lieu of single-molecule experiments.

Our results indicate that kinetic studies do not exhibit the same degree of correlation. Mechanical denaturation resulted in statistically significant increases in the rates of both unfolding and refolding compared to chemical denaturation. This could be simply due to a stochastic difference since chemical denaturation is generally used for bulk refolding experiments using tools like intrinsic fluorescence. In these types of experiments, the measured rate is the average of all the molecules in the reaction compared to the rate determined from a single molecule. Alternatively, this difference may reflect a difference in the unfolded state, changing either the path or distance required to refold. Our current data cannot distinguish between these two possibilities.

Our model system, the I83 immunoglobulin (Ig) domain from titin, has been shown to be relatively unstable compared to most other Ig domains in titin but it is stabilized by the presence of calcium. Mechanical unfolding and refolding experiments conducted in the presence of calcium as part of this study correlated with our previous results under lower forces. Mechanically induced unfolding rates measured in the presence of calcium exhibited a statistically significant decrease in unfolding rate for forces ≤40 pN (Figure 3), as well as a significant shift in the fraction folded at 10 pN (Figure 4). Interestingly, this effect was not statistically significant when unfolding forces were >40 pN and was similarly lost at temperatures > 60 °C and at urea concentrations >4.0 M (Figure 1). This suggests that calcium is stabilizing a conformation that exists at lower forces, denaturant concentration, or temperature but that this conformation is lost at higher forces, temperatures, and denaturant conditions.

### 4.2. Mechanistic Role for Calcium Binding to I83 Domain

The N2A region of titin has become a region of particular interest in recent years as it has become apparent that this region is a signaling hub for a number of regulatory proteins, including the Muscle Ankryin Repeat proteins (MARPs), the calpain-3 protease, the SET and MYND-containing lysine methyl transferase 2 (Smyd2) and actin [52,53,54,55,56,57]. The N2A region is composed of four Ig domains and the I83 domain used in this study is the terminal domain in this region. The I83 domain’s contribution to the N2A region’s functions is of interest since this domain contains one of the calpain-3 binding sites in the N2A region [53,58]. A portion of the I83 domain is also deleted in *mdm* mice, a model system that exhibits altered titin behavior [54,59]. An increased understanding of the function of the I83 domain would help with the development of a more comprehensive model of the function of the N2A region overall.

It is interesting that the I83 domain has the lowest stability given that binding of calpain-3 occurs in this region. It is possible that the domain is stabilized by binding of calpain-3 and that this interaction helps to regulate titin function, similar to how CARP binding to I81 stabilizes the interaction between N2A and actin [56,60]. The loss of titin function in active muscle in *mdm* mice also points to the importance of this region, especially since recent studies have shown the deletion of the regions of the PEVK that are lost in *mdm* mice does not reproduce the *mdm* phenotype [61]. Taken together, it is not unreasonable to conclude that there is a functional significance to the low stability of this domain, and therefore calcium binding to I83 has a functional significance as well.

The canonical calcium binding site is an EF-hand motif [62], which does not exist in Ig domains. However, our NMR and mutagenesis studies have shown that there is a non-canonical binding site in the FG-loop, along the I82-I83 interface [4]. Based on the binding affinity of calcium for this site, it is hypothesized that binding likely only occurs in an activated muscle, leading to the speculation that this represents a regulatory mechanism for an I83 function in active muscle that is not necessary in resting muscle. This could be related to calpain-3 binding, actin binding or both. The loss of calcium-induced stabilization at higher forces adds an intriguing element to this model. One potential model is that the binding of calcium to I83 when muscle is activated produces a binding site for calpain-3 to prevent activation of stress-response pathways. However, when a muscle is stretched to the point of damage, this would overcome the calcium stabilization and result in unfolding of I83, releasing calpain-3 and activating stress response pathways. There are a number of points of speculation in this model and more work is necessary to understand this region, but the mechanical force studies reported here provide some intriguing potential new possibilities.

### 4.3. Force Unfolding of Physiological Proteins including Ig-like Domains in Titin

Immunoglobulin domains are highly conserved structural elements used as building blocks by many multi-domain proteins, from cellular receptors to immune system proteins to mechanical/elastic proteins like titin [63,64,65,66,67]. Titin’s extensible I-band of skeletal titin consists of ~90 Ig-like domains [68] that share highly conserved structures [69]. Only a subset of the titin Ig domains have been biophysically characterized but it is well-established that a range of stabilities exist between I-band Ig domains [27,43]. What remains poorly understood is the functional significance of these differences. The long-standing model of titin extension starts with the straightening of the I-band Ig-domains as the muscle is lengthened. The application of low force disrupts the domain-domain interactions between Ig-domains resulting in a more extended titin conformation [49]. As more force is applied to stretch the muscle further, the disordered PEVK region begins to stretch, resulting in more extended conformations and greater passive tension [70,71,72].

The role of Ig-domain unfolding in passive tension has been debated for a number of years. The force required to unfold the Ig domains studied in early AFM experiments were larger than could be obtained physiologically and therefore it was questioned whether Ig domains would become unfolded during eccentric contractions [73]. Recent studies using instruments with lower force ranges than AFM can obtain have demonstrated that spontaneous unfolding and refolding of Ig domains can occur at physiologically relevant forces [30,43], especially when physiological temperatures (~37 °C) are applied [74]. These new results suggest that Ig-domain folding and unfolding may actually have a physiological relevance that needs to be explored further. The diversity of Ig domains in titin provides a rich toolbox to explore this question but it would be a Herculian undertaking to measure stability of all 90+ domains using magnetic tweezers or other single-molecule techniques. However, the results from this study indicate that high-throughput denaturation studies could be used to classify the various domains based on equilibrium stability measurements and then a subset of domains with representatives from each group could be studied by single-molecule techniques with confidence in the reliability of the classification.

## 5. Conclusions

Protein folding is important in a variety of physiological processes, including the impact on the extension of muscle proteins. We found that thermal, chemical, and force unfolding yielded similar ΔG_unfolding_ values, while slightly different rate constants were observed between chemical and force refolding experiments. Interestingly, the rate of both unfolding and refolding was faster for force-induced unfolding and refolding. This implies that the act of tethering may increase the rate of transition between the folded and unfolded state. This is important to other biological processes where the protein might be tethered, like folding on the ribosome during protein synthesis or unfolding/refolding events in elastic proteins like titin. Recent findings suggest that the folding mechanism of a newly synthesized protein while tethered to a ribosome can influence the final folded structure in vivo [75]. Most protein folding studies do not take into account the impact of tethering on protein-folding. Since many proteins begin folding while still tethered to the ribosome, more physiologically accurate measurements of protein folding following synthesis can be made using a tether protein in a magnetic tweezer system. Molecular tweezers have demonstrated to be a reliable tool for studying the folding of tethered molecules and can continue to provide further insight into physiologically relevant protein folding.

## Figures and Tables

**Table 1:** Free energy values indicated similar values for thermal and chemical unfolding and refolding.

	Thermal	Chemical
ΔG_unfolding_ (kcal/mol) Ca-free	3.2 ± 0.1	3.3 ± 0.3
ΔG_refolding_ (kcal/mol) Ca-free	3.1 ± 0.2	3.4 ± 0.04
ΔG_unfolding_ (kcal/mol) pCa 4.3	4.2 ± 0.1	4.8 ± 0.1

**Table 2:** Rate constants and free energy values indicated stabilization by calcium in both chemical and force unfolding.

	Chemical		Force	
	Ca-Free	pCa 4.3	Ca-Free	pCa ≤ 4
Natural Log of Unfolding Rate Ln k_u_	−5.52 ± 0.10	−6.04 ± 0.08	−5.01 ± 0.34	−5.68 ± 0.21
Unfolding Rate k_u_ (s^−1^)	0.004 ± 0.0004	0.0024 ± 0.0002	0.0067 ± 0.0019	0.0034 ± 0.0006
Natural Log of Refolding Rate Ln k_f_	−0.013 ± 0.15	0.13 ± 0.14	1.00 ± 0.28	1.20 ± 0.23
Refolding Rate K_f_ (s^−1^)	0.99 ± 0.13	1.14 ± 0.15	2.72 ± 0.66	3.32 ± 0.68
ΔG_unfolding_ Equation (7) (kcal/mol)	3.3 ± 0.25	3.7 ± 0.22	3.5 ± 0.62	4.0 ± 0.44

## Data Availability

All raw data is available upon request.
